# Comparison between standard Vs. Escalated dose venous thromboembolism (VTE) prophylaxis in critically ill patients with COVID-19: A two centers, observational study

**DOI:** 10.1016/j.jsps.2022.01.022

**Published:** 2022-02-03

**Authors:** Ohoud Aljuhani, Khalid Al Sulaiman, Awatif Hafiz, Khalid Eljaaly, Aisha Alharbi, Rahmah Algarni, Sarah Al Homaid, Khawla Kahtani, Tareq Alsulaiman, Ramesh Vishwakarma, Ghassan Al Ghamdi, Mai Alalawi, Ghazwa B. Korayem

**Affiliations:** a Department of Pharmacy Practice, Faculty of Pharmacy, King Abdulaziz University, Jeddah, Saudi Arabia; b Pharmaceutical Care Department, King Abdulaziz Medical City, Riyadh, Saudi Arabia; c College of Pharmacy, King Saud bin Abdulaziz University for Health Sciences, Riyadh, Saudi Arabia; d King Abdullah International Medical Research Center, Biostatistics and Bioinformatics Department, Riyadh, Saudi Arabia; e College of Pharmacy, University of Arizona, Tucson, AZ, United States; f Pharmaceutical Care Department, King Abdulaziz University Hospital, Jeddah, Saudi Arabia; g Department of Orthopedic Surgery, Imam Abdulrahman Al Faisal Hospital, Riyadh, Saudi Arabia; h College of Medicine, King Saud Bin Abdulaziz University for Health Sciences, King Abdullah International Medical Research Center, Saudi Arabia; i Intensive Care Department, King Abdulaziz Medical City, Riyadh, Saudi Arabia; j Fakeeh College of Medical Sciences, Jeddah, Saudi Arabia; k Department of Pharmacy Practice, College of Pharmacy, Princess Nourah bint Abdulrahman University, Riyadh, Saudi Arabia; l Statistics Department, European Organization for Research and Treatment of Cancer (EORTC) Headquarters, Brussels, Belgium

**Keywords:** COVID-19, SARS-Cov-2, VTE prophylaxis, Escalated-dose, Standard dose, Enoxaparin, Heparin, Critically ill, Intensive Care Units (ICUs), ICUs, Intensive care units, COVID-19, Coronavirus disease, VTE, Venous thromboembolism, MV, Mechanical ventilation, LOS, Length of Stay

## Abstract

**Introduction:**

The risk of mortality in patients with COVID-19 was found to be significantly higher in patients who experienced thromboembolic events. Thus, several guidelines recommend using prophylactic anticoagulants in all COVID-19 hospitalized patients. However, there is uncertainty about the appropriate dosing regimen and safety of anticoagulation in critically ill patients with COVID-19. Thus, this study aims to compare the effectiveness and safety of standard versus escalated dose pharmacological venous thromboembolism (VTE) prophylaxis in critically ill patients with COVID-19.

**Methods:**

A two-center retrospective cohort study including critically ill patients aged ≥ 18-years with confirmed COVID-19 admitted to the intensive care unit (ICU) at two tertiary hospitals in Saudi Arabia from March 1st, 2020, until January 31st, 2021. Patients who received either Enoxaparin 40 mg daily or Unfractionated heparin 5000 Units three times daily were grouped under the “standard dose VTE prophylaxis and patients who received higher than the standard dose but not as treatment dose were grouped under ”escalated VTE prophylaxis dose“. The primary outcome was the occurance of thrombotic events, and the secondary outcomes were bleeding, mortality, and other ICU-related complications.

**Results:**

A total of 758 patients were screened; 565 patients were included in the study. We matched 352 patients using propensity score matching (1:1). In patients who received escalated dose pharmacological VTE prophylaxis, any case of thrombosis and VTE were similar between the two groups (OR 1.22;95 %CI 0.52–2.86; *P* = 0.64 and OR 0.75; 95% CI 0.16–3.38; *P* = 0.70 respectively). However, the odds of minor bleeding was higher in patients who received escalated VTE prophylaxis dose (OR 3.39; 95% CI 1.08–10.61; *P* = 0.04). There was no difference in the 30-day mortality nor in-hospital mortality between the two groups (HR 1.17;95 %CI0.79–1.73; *P* = 0.43 and HR 1.08;95 %CI 0.76–1.53; *P* = 0.83, respectively).

**Conclusion:**

Escalated-dose pharmacological VTE prophylaxis in critically ill patients with COVID-19 was not associated with thrombosis, or mortality benefits but led to an increased risk of minor bleeding. This study supports previous evidence regarding the optimal dosing VTE pharmacological prophylaxis regimen for critically ill patients with COVID-19.

## 1 Introduction

Coronavirus disease 2019 (COVID-19) is an infectious disease causing a serious global pandemic (Guan et al., 2020). The clinical presentation of infected patients is heterogeneous, ranging from asymptomatic to severe pneumonia accompanied with complications such as respiratory failure, leading to mechanical ventilation (MV) and intensive care unit (ICU) admission, or death (Guan et al., 2020). Several risk factors were associated with increased mortality of hospitalized patients with COVID-19 (Zhou et al., 2020). Factors include elevated D-dimer level (>1mcg/ml), older ages, cardiovascular diseases, diabetes mellitus, obesity, and high baseline sequential organ failure assessment (SOFA) score(Zhou et al., 2020). In critically ill patients, respiratory failure, acute kidney injury, and thrombosis are common complications (al Sulaiman et al., 2021). Many of these complications in critically ill patients are attributed to the severe phase of host inflammatory response (Lu et al., 2021, Zhang et al., 2020). This reponse caused by excess host cytokines release known as “cytokine release syndrome,” which causes capillary damage, thrombosis, and multiorgan dysfunction (Eljaaly et al., 2021, Rad et al., 2021, Zhang et al., 2020).

Although COVID-19 infection targets mainly the respiratory system, several organs can be predisposed, including the vascular system (Wang et al., 2020a). A recently published *meta*-analysis that included forty-two studies reported the rates of venous thromboembolism (VTE) in critically ill COVID-19 patients were remarkably higher than non-critical patients, reaching 31% (Malas et al., 2020). Moreover, the risk of mortality with COVID-19 was significantly higher in patients who experienced thromboembolic events (Malas et al., 2020). Thus, several guidelines, including the International Society of Thrombosis and Hemostasis (ISTH), the American Society of Hematology (ASH), and the American College of Chest Physicians (CHEST), recommend the use of prophylactic anticoagulants in all hospitalized patients with COVID-19 (whether critically ill or not) unless there is a contraindication or evidence of increases the risk of bleeding (Cuker et al., 2021, Moores et al., 2020, Spyropoulos et al., 2020).

Dosing of anticoagulation prophylaxis in critically ill patients with COVID-19 remains a considerable controversy. Currently prescribed anticoagulation regimes rely more on physicians' decisions and expert opinions. Besides, some experts push for escalating anticoagulant doses due to the severity of the disease in ICU patients (Malas et al., 2020). Prescribing the intermediate or high dose regimen of VTE prophylaxis in patients with COVID-19 was more common early in the pandemic when evidence emerged about the abnormal coagulation laboratory results and related mortality in patients with COVID-19 pneumonia (Helms et al., 2020, Tang et al., 2020). In addition, many studies reported thrombotic complications despite anticoagulation use supporting the use of escalated doses of anticoagulation for VTE prophylaxis in critically ill patients (Helms et al., 2020, Jonmarker et al., 2020). Therefore, several national and international practice protocols recommended dose escalation of VTE thromboprophylaxis in patients with COVID-19 and elevated coagulation markers such as the D-dimer (Barnes et al., 2020, Marietta et al., 2020, Saudi Ministry of Health, 2021a, Saudi Ministry of Health, 2020). Later, much evidence emerged about the adverse outcomes or no additional benefits of intermediate and treatment anticoagulation doses in critically ill patients (ATTACC Investigators et al., 2021).

Nonetheless, several studies issued after that continue to support the use of high doses of thromboprophylaxis (Lavinio et al., 2021, Tacquard et al., 2021). Other trials are still ongoing to help determine the optimal dose of anticoagulants in critically ill COVID-19 patients (Neal Mathew, n.d.). There is uncertainty about the appropriate dosing regimen, safety, and the predisposing factors for thrombosis or bleeding risk in critically ill patients with COVID-19. Thus, the study aims to compare the effectiveness and safety of standard and escalated dose pharmacological VTE prophylaxis in critically ill patients with COVID-19.

## 2 Methods

### 2.1 Study design and population

A two-center retrospective cohort study was conducted, including critically ill patients aged ≥ 18-years with confirmed COVID-19 who were admitted to the ICUs of two tertiary hospitals in Saudi Arabia from March 1st, 2020, until January 31st, 2021. Patients were excluded if they were not on pharmacological VTE prophylaxis, on lower than standard dose VTE prophylaxis (i,e, Enoxaparin < 40 mg/day or Unfractionated heparin (UFH) < 5000 Units three times /day). Also excluded if received treatment dose of anticoagulation for other indication (s) (e.g., Atrial fibrillation.), active bleeding within 24 h of ICU admission, have platelets count < 50,000 10^9^/L and or ICU length of stay (LOS) ≤ One day. Data was obtained from the patients' medical records at King Abdulaziz University Hospital, Jeddah, and King Abdulaziz Medical City, Riyadh.

Patients were classified into two groups based on the VTE prophylaxis dosing intensity (Standard vs. escalated dose) during the ICU stay. Patients who received either enoxaparin 40 mg daily or UFH 5000 Units three times daily were grouped under the “standard dose VTE prophylaxis”(Cuker et al., 2021, Moores et al., 2020). While patients who received a higher than standard dose but not as treatment dose (enoxaparin 1 mg/kg q12hr or 1.5 mg/kg q24hr or UFH infusion) were categorized as receiving “Escalated VTE prophylaxis dose”. The pharmacological thromboprophylaxis dose is usually decided based on physicians' judgment who usually follow available evidence and the Saudi Ministry of Health (MOH) protocol for patients with COVID-19 (Saudi Ministry of Health, 2021a, Saudi Ministry of Health, 2020). During the study period, the MOH protocol recommended to give a standard dose of enoxaparin for the patient with D-dimer < 1 mcg/mL if the patient's weight is < 100 kg. While recommends higher doses of (>40 mg daily enoxaparin) if the patients d-dimer > 1 mcg/mL or if their weight above 100 kg (Saudi Ministry of Health, 2021a, Saudi Ministry of Health, 2020). Patients were followed during their ICU stay. The study was approved by King Abdulaziz University Hospital (Reference # 554–20) and King Abdullah International Medical Research Center (KAIMRC)  (Reference # NRC21R-189-04).

### 2.2 Data collection

We collected demographic data, comorbidities, vital signs, laboratory tests, baseline severity scores (i.e. Acute Physiology and Chronic Health Evaluation II (APACHE II), Sequential Organ Failure Assessment (SOFA)), Nutrition Risk in Critically ill (NUTRIC) scores, PADUA score, Glasgow Coma Score (GCS). Additionally, acute kidney injury, fluid balance, mechanical ventilation (MV) needs and MV parameters (e.g., PaO2/FiO2 ratio, Fio2 requirement) and inflammatory markers (CRP, procalcitonin) within 24 hours of ICU admission. Also, renal profile, liver function tests (LFTs), coagulation profile (i.e., INR, aPTT, fibrinogen) within 24 hours of ICU admission were collected. During ICU stay, radiological findings (using Ultrasound or CT scan as appropriate), major or minor bleeding data, and RBCs transfusion were recorded for the eligible patients. All patients were followed until they were discharged from the hospital or died during the in-hospital stay, whichever occurred first.

### 2.3 Outcomes

To evaluate the effectiveness of two VTE pharmacological prophylaxis regimens, we used the primary endpoint: VTE or any thrombotic events during ICU. Both VTE or any thrombotic event during ICU stay were identified using the International Classification of Diseases (ICD), 10th Revision, Clinical Modification (ICD10-CM) code (“ICD - ICD-10-CM - International Classification of Diseases, Tenth Revision, Clinical Modification,” 2021). The secondary endpoints assessing safety outcomes were the ICU-related complication (s) during the ICU stay (i.e., major bleeding, minor bleeding, RBC transfusion during ICU stay, respiratory failure requiring MV). In addition, follow-up outcomes include hospital LOS, ICU LOS, MV duration, 30-day, and in-hospital mortality.

ICU-related complications were identified if the patient experienced major bleeding according to the International Society on Thrombosis and Hemostasis (ISTH) definition of major bleed (SCHULMAN and KEARON, 2005). Any patient not fulfilling the criteria of major or clinically significant bleeding was identified as having a minor bleed. Acute kidney injury (AKI) was defined using kidney disease: Acute Kidney Injury Network (AKIN) definition (Lin, 2012). Respiratory failure was defined as either hypoxemic respiratory failure (PaO_2_ < 60 mm Hg with a normal or low arterial carbon dioxide tension (PaCO_2_) or hypercapnic respiratory failure (PaCO_2_ > 50 mm Hg) that requires invasive mechanical ventilation.

### 2.4 Statistical analysis

We presented continuous variables as mean and standard deviation (SD), or median and interquartile range (IQR), and categorical variables as number (percentage) as appropriate. The normality assumptions were assessed for all numerical variables using a statistical test (i.e., Shapiro–Wilk test) and graphical representation (i.e., histograms and Q-Q plots). We compared categorical variables using the chi-square or Fisher exact test. We compared the normally distributed continuous variables using unpaired student *t*-test and other non-normally distributed continuous variables with the Mann-Whitney *U* test. Baseline characteristics, baseline severity, and outcome variables were compared between the two groups.

Propensity score matching procedure (Proc PS match) (SAS, Cary, NC) was used to match patients who received escalated dosing (Active) to patients who received standard dosing VTE prophylaxis regimen (control) according to severity score (APACHE II and SOFA score), history of chronic kidney disease (CKD), and AKI within 24 hours of ICU admission. A greedy nearest neighbor matching method was used in which one patient in the active group was matched with each patient in the control group. This eventually produces the smallest within-pair difference among all available pairs with treated patients. These patients are matched only if the difference in the logits of the propensity scores for pairs of patients from the two groups is less than or equal to 0.5 times the pooled estimate of the standard deviation.

Model fit was assessed using the Hosmer-Lemeshow goodness-of-fit test. Multivariable Cox proportional hazards regression analyses were performed for 30-day, and in-hospital mortality and Kaplan-Meier (KM) plots were generated for these outcomes. Multivariable regression analysis and negative binomial regression were used after adjusting for the severity score (APACHE II and SOFA score), history of chronic kidney disease (CKD), and acute kidney injury (AKI) within 24 h of ICU admission. The odds ratios (OR), hazard ratio (HR), or estimates with the 95% confidence intervals (CI) were reported as appropriate. No imputation was made for missing data as the cohort of patients in our study was not derived from random selection. We considered a P value of < 0.05 statistically significant and used SAS version 9.4 for all statistical analyses.

## 3 Results

**Fig. 1:**
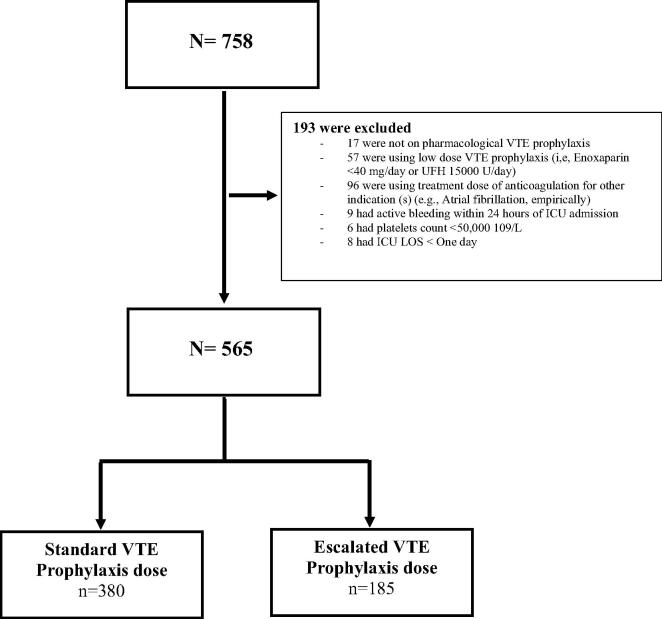
Flowchart of adult patients admitted to the intensive care unit (ICU) with confirmed COVID-19.

Among the 758 patients initially screened, a total of 565 patients were included in the study who received pharmacological VTE prophylaxis at ICU admission (Fig. 1). The patient characteristics and demographic information are described in Table 1. The escalated-dose regimen of pharmacological VTE prophylaxis was given to 185 patients, whereas 380 patients received a standard dose regimen. We matched 352 patients using propensity score matching (1:1) according to the baseline severity scores, history of CKD, and AKI within 24 h of ICU admission. We observed that all included patients received early pharmacological VTE prophylaxis within 24 h of ICU admission.

**Table 1:** Baseline characteristics of the patients admitted to the intensive care unit (ICU) with COVID-19 using anticoagulation.

	**Before Propensity score Matching**				**After Propensity score matching**			
	**Overall (565)**	**Standard dose** **(N = 380)**	**Escalated dose** **(N = 185)**	***P*-value**	**Overall (352)**	**Standard dose** **(N = 176)**	**Escalated dose** **(N = 176)**	***P* value**
**Age (Years), Mean (SD)**	60.9 (14.42)	61.2 (14.48)	60.4 (14.32)	0.3150^	59.2 (13.89)	58.6 (14.33)	59.9 (13.44)	0.4947^
**Gender – Male, n (%)**	404 (72.3)	271 (71.7)	133 (73.5)	0.6587^^	250 (72.3)	123 (70.7)	127 (73.8)	0.5132^^
**Weight (kg), Mean (SD)**	82.3 (18.78)	(81.3 (17.56)	84.4 (20.99)	0.3166^	82.9 (20.50)	81.2 (19.65)	84.7 (21.24)	0.2716^
Body Mass Index (oBdy**BMI), Mean (SD)**	31.1 (8.61)	30.9 (8.67)	31.6 (8.49)	0.3527^	31.0 (7.73)	30.3 (6.62)	31.7 (8.67)	0.3740^
**Heart Rate (HR), Median (**Q1, Q3**)**	103.0 (91, 115.00)	104.0 (91.00, 115)	101.5 (90.50, 113)	0.3265^	104.0 (91, 115)	106.0 (93, 117)	101.0 (91, 112)	0.0854^
**Respiratory Rate (RR/minute) Median, (Q1, q3)**	30.0 (24, 35.00)	30.0 (24, 35)	30.0 (25, 35)	0.4560^	30.0 (25, 35)	31.0 (24, 35)	30.0 (25, 35)	0.5606^
**Maximum body temperature(°C), Median, (Q1, Q3)**	37.4 (37, 38.30)	37.4 (37, 38.30)	37.4 (37, 38.10)	0.0987^	37.4 (37, 38.30)	37.4 (37, 38.30)	37.4 (37, 38.10)	0.2069^
**MV during ICU stay within 24hr, n (%)**	356 (6 4)	247 (65.7)	109 (60.6)	0.2377^^	206 (59.4)	101 (58.0)	105 (60.7)	0.6156^^
**A-**A **Gradient, Median (Q1, Q3)**	411.9 (241.60, 568.80)	418.0 (232.45, 570.40)	401.6 (253.80, 556.80)	0.6488^	411.2 (268.80, 565.50)	414.1 (249.10, 565.60)	410.3 (284.10, 560.50)	0.9846^
**PaO2/FiO2 ratio within 24 h of admission, Median (Q1, Q3)**	81.2 (61.30, 118.30)	79.6 (62, 122.60)	82.5 (61.11, 115)	0.7451^	81.2 (60.62, 114.00)	77 (59.90, 115)	81.6 (61.11, 112.70)	0.8441^
**GCS Baseline, Median (Q1, Q3)**	15(13., 15)	15 (11, 15)	15 (15, 15)	0.0004^	15 (15. 15.00)	15 (15, 15)	15.0 (15, 15)	0.2657^
**APACHE II score, median (Q1, Q3)**	12 (7, 22)	12 (7, 24.)	11 (7, 17)	0.0124^	11.0 (7, 16)	11.0 (7, 15	11.0 (7, 17)	0.9036^
**SOFA score, Median (Q1, Q3)**	4.0 (2, 7)	5.0 (3, 8)	4.0 (2, 6)	0.0014^	4.0 (2, 6)	4.0 (2.00, 6.)	4.0 (2, 6)	0.7683^
**NUTRIC Score, Median (Q1, Q3)**	3.0 (2, 5)	3.0 (2, 6)	3.0 (2, 4)	0.0021^	3.0 (2.00, 4.00)	3.0 (1.00, 4.00)	3.0 (2, 4)	0.6419^
**PADUA Score, Median (Q1, Q3)**	5.0 (4.00, 6.00)	5.0 (4.00, 6.00)	5.0 (4, 6)	0.8668^	5.0 (4, 5.)	5.0 (4.00, 5.00)	5.0 (4, 6)	0.6742^
**Serum creatinine, Median (Q1, Q3)**	86.0 (69.00, 126.00)	92.0 (71, 142)	78.0 (66.50, 97.50)	<0.0001*	78.0 (65, 101.)	75.5 (64, 103)	78.0 (67, 98)	0.8480^
Estimated Glomerular Filtration Rate (eGFR)**, Median (Q1, Q3)**	79.0 (48.00, 100.00)	73.5 (40.50, 97.00)	86.0 (68, 104)	<0.0001*	86.0 (65.00, 105.00)	87.0 (63.00, 106.00)	85.0 (67.00, 104.00)	0.6926*
**Acute Kidney Injury (AKI) within 24 h of ICU admission, n (%)**	143 (25.7)	120 (31.9)	23 (12.7)	<0.0001^^	44 (12.7)	21 (12.1)	23 (13.3)	0.7315^^
**Blood urea nitrogen (BUN), Median (Q1, Q3)**	7.1 (4.80, 11.75)	7.5 (5, 15.10)	6.6 (4.70, 8.90)	0.0011^	6.5 (4.60, 9.30)	6.3 (4.40, 9.80)	6.6 (4.70, 8.90)	0.8286^
**Alanine aminotransferase (**ALT**), Median (Q1, Q3)**	39 (26.00, 66.00)	38 (25, 64)	40(27, 66)	0.8947^	38 (26, 66)	37 (24, 66)	39.5 (27.00, 66.00)	0.7559^
**Aspartate aminotransferase (**AST**), Median (Q1, Q3)**	55.0 (36, 78)	55.0 (36, 81)	52.0 (34., 75.00)	0.1782^	53.0 (34, 76.)	54.0 (3, 77)	51.5 (34, 73)	0.4898^
**C-ReactiveProtein(mg/l)m Median (Q1, Q3)**	156.0 (84, 231)	154.5 (86.00, 231)	158.0 (80, 234)	0.8411^	155 (80.50, 229.50)	152. (76.50, 233.50)	157.5 (83, 225.50)	0.5606^
**Fibrinogen Level (gm**/**l), Median (**Q1, Q3**)**	6.5 (4.95, 8.80)	6.9 (5.01, 287)	5.8 (4.66, 7.83)	0.0439^	6.0 (4.54, 7.78)	6.5 (4.49, 7.91)	5.8 (4.85, 7.73)	0.5627^
**D-dimer (mg/l), Median (Q1, Q3)**	1.1 (0.67, 2.54)	1.1 (0.67, 2.78)	1.2 (0.67, 2.41)	0.8156^	1.0 (0.61, 1.96)	0.9 (0.59, 1.86)	1.1 (0.61, 2.25)	0.2251^
**Total WBC, Median (Q1, Q3)**	9.6 (6.90, 13.05)	10.0 (6.95, 13.60)	9.4 (6.86, 12.60)	0.1519^	9.4 (6.82, 12.90)	10.0 (6.81, 13.60)	9.1 (6.85, 12.50)	0.1728^
**Hematocrit (Hct), Median (Q1, Q3)**	0.4 (0.36, 0.46)	0.4 (0.35, 0.47)	0.4 (0.37, 0.44)	0.7375^	0.4 (0.36, 0.44)	0.4 (0.35, 0.44)	0.4 (0.37, 0.44)	0.1375^
**Platelets count, Median (Q1, Q3)**	256 (200, 330.50)	260 (204, 333)	254.0 (193, 321)	0.6296^	256.0 (203, 331)	264.0 (210, 336)	254 (193, 318)	0.2615^
**International normalized ratio (INR), Median (Q1, Q3)**	1.1 (1.03, 1.17)	1.1 (1.03, 1.18)	1.1 (1.02, 1.15)	0.2666^	1.1 (1.03, 1.14)	1.1 (1.03, 1.14)	1.1 (1.03, 1.15)	0.7273^
**aPTT, Median (Q1, Q3)**	29.5 (26.40, 32.60)	29.9 (26.70, 33.00)	28.5 (25.65, 31.50)	0.0032^	29.0 (26.00, 32.00)	29.5 (26.30, 32.30)	28.5 (25.70, 31.50)	0.1519^
**Systemic Corticosteroids use during ICU, n (%)**	496 (89.5)	329 (88.0)	167 (92.8)	0.0833^^	313 (90.7)	154 (89.0)	159 (92.4)	0.2729^^
**Tocilizumab Use, n (%)**	194 (34.9)	125 (33.4)	69 (37.9)	0.2973^^	130 (37.8)	64 (37.4)	66 (38.2)	0.8900^^

*T Test/^ Wilcoxon rank sum test is used to calculate the P-value.

^^ Chi square/** Fisher’s Exact teat is used to calculate P-value.

### 3.1 Patients’ characteristics

Most of the patients in both groups were men (72.3%), and the mean age of the patients was 60.9 ± 14 years. Severity scores (i.e., APACHE II and SOFA scores), procalcitonin levels, fibrinogen levels, serum creatinine, acute kidney injury within 24 hours of ICU admission were higher in patients who received standard dosing of pharmacological VTE prophylaxis as shown in Table 1. The most common comorbidities in both groups were diabetes mellitus at 60%, hypertension at 56%, and dyslipidemia at 23%. Moreover, CKD and history of VTE were higher in the standard-dose regimen compared with the escalated-dose pharmacological VTE prophylaxis regimen, as demonstrated in the **additional file 1: Table S1.** There were no significant differences in baseline characteristics after adjustment using propensity score matching.

### 3.2 Primary outcome

The occurance of VTE among the escalated-dose regimen was lower at 1.7% compared to the standard dose (2.27%). VTE was less likely to occur in patients who received escalated dose pharmacological VTE prophylaxis (OR 0.75; 95% CI 0.16–3.38; *P* = 0.70), while any case of thrombosis during ICU was more likely to occur (OR 1.22;95 %CI 0.52–2.86; *P* = 0.64). However, both were not statistically significant compared with the standard dose regimen, as presented in Table 2.

**Table 2:** Multivariate regression analysis for ICU complication (s) during ICU stay.

**Outcomes**	**VTE prophylaxis**		***P* value** ^^	**Odds Ratio (OR) (95 %CI)**	***P* value**^$^
	**Escalated**	**Standard dose**			
**Venous Thromboembolism (VTE), n/N(%)**	3/176 (1.7)	4/176 (2.27)	0.70	0.75 (0.16, 3.38)	0.70
**Any Thrombosis During ICU, n/N(%)**	13/172 (7.5)	11/175 (6.2)	0.64	1.22 (0.52, 2.86)	0.64
**Major bleeding, n/N(%)**	9/174 (5.2)	7/171 (4.09)	0.63	1.28 (0.46, 3.53)	0.63
**Requiring RBCs transfusion during ICU stay, n/N(%)**	23/165 (13.9)	29/173 (16.7)	0.47	0.80 (0.44, 1.46)	0.46
**Minor bleeding, n/N(%)**	13/173 (7.5)	4/171 (2.3)	0.03	3.39 (1.08, 10.61)	0.04
**Respiratory Failure Required MV, n/N (%)** $*	23/66 (34.8)	26/73 (35.6)	0.92	0.98 (0.49, 1.96)	0.95
**New Onset Afib., n/N(%)**	16/162 (9.9)	17/163 (10.4)	0.87	0.94 (0.457, 1.938)	0.87
**ICU readmission within 3 months, n/N(%) ¥**	10/103 (9.7)	13/112 (11.6)	0.65	0.81 (0.34, 1.95)	0.63

**-** Denominator of the percentage is the total number of patients.

**^^** Chi-square test is used to calculate the P-value.

$ Propensity score is used to calculate Odds ratio and p-value.

$* Denominator of the percentage is non-mechanically ventilated patients with 24 h of ICU admission.

### 3.3 Secondary outcomes

#### 3.3.1 ICU complications

In patients who received escalated-dose pharmacological VTE prophylaxis, minor bleeding was higher by 3.5 folds than standard-dose pharmacological VTE prophylaxis (OR 3.39; 95% CI 1.08–10.61; *P* = 0.04). The proportion of minor bleeding was 2.3% in the patients who received standard dose compared with 7.5% in patients who received escalated-dose pharmacological VTE prophylaxis. In terms of major bleeding and RBC s transfusion requirement during ICU stay, there are no statistical differences between the two groups ((OR 1.28; 95% CI 0.46–3.53; *P* = 0.63) and (OR 0.80; 95% CI 0.44–1.46; *P* = 0.46), respectively as shown in Table 2.

**Table 3:** Multivariate regression analysis for follow-up outcomes.

**Outcomes**	**Crude Analysis**		***P* value**	**Hazard Ratio (95 %CI)**	***P* value** $
	**Standard dose**	**Escalated dose**			
**In-hospital mortality, n/N(%)** Δ	64/173 (37.0)	65/171 (38.0)	0.85^^	1.08 (0.76, 1.53)	0.67
**30-day mortality, n/N(%)** Δ	48/173 (27.7)	54/172 (31.4)	0.46^^	1.17 (0.79, 1.73)	0.43
				**Beta- coefficient (95 %CI)**	**P-value** $*
**ICU Length of Stay(Days), Median (**Q1, Q3**) ^&^**	9.0 (5.0,15.0)	8.0 (5.0,13.0)	0.39^	−0.12 (-0.31, 0.07)	0.23
**Hospital Length of Stay(Days), Median (Q1, Q3) ^&^**	17.0 (11.0,24.0)	14.0 (10.0,24.0)	0.22^	−0.11 (-0.27, 0.06)	0.20
MV duration (Days), Median (Q1, Q3) &	6.0 (1.0, 13.0)	3.0 (0.0, 10.0)	0.29^	-0.17 (-0.58, 0.24)	0.41

Δ The Denominator is the total number of patients

**&** Denominator is the number of patients who survived.

^ Wilcoxon rank-sum test is used to calculate the P-value.

^^ Chi-square test is used to calculate the P-value.

$* Propensity score is used to calculate Beta- coefficient (estimate) and p-value.

$ Propensity score is used to calculate hazard ratio and p-value.

#### 3.3.2 Follow-up outcomes and mortality

During hospitalization, the proportion of patients who died in the standard dose regimen group was 37% compared to 38% in escalated-dose VTE pharmacological prophylaxis, as demonstrated in table 3. In the multivariable cox analysis, there was no significant difference in the 30-day mortality nor in-hospital mortality between the two groups (HR 1.17; 95 %CI 0.79–1.73*P* = 0.43) and (HR 1.08 95 %CI 0.76–1.53*P* = 0.83) respectively. The overall survival probabilities were similar during hospital stay between the two groups before and after propensity score matching as depicted in Fig. 2a, Fig. 2b**.** No differences were observed between the two dosing regimens in ICU LOS (Beta coefficient = -0.12; CI −0.31, 0.07; *P* = 0.23), hospital LOS (Beta coefficient = -0.11; CI −0.27, 0.06; *P* = 0.20) and MV duration  (Beta coefficient = -0.17; CI −0.58, 0.24; *P* = 0.41)  Table 3.

**Fig. 2a:**
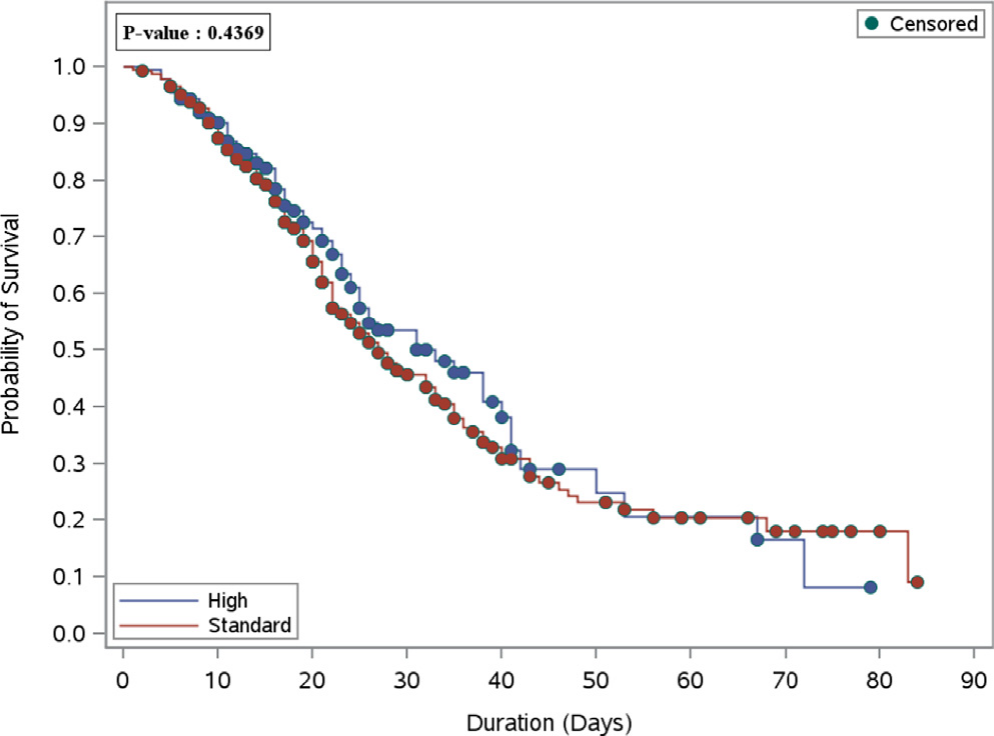
Kaplan Meier Curve for overall survival comparing the escalated "High" dose and standard dosing of VTE prophylaxis during ICU stay - Before Propensity score matching.

**Fig. 2b:**
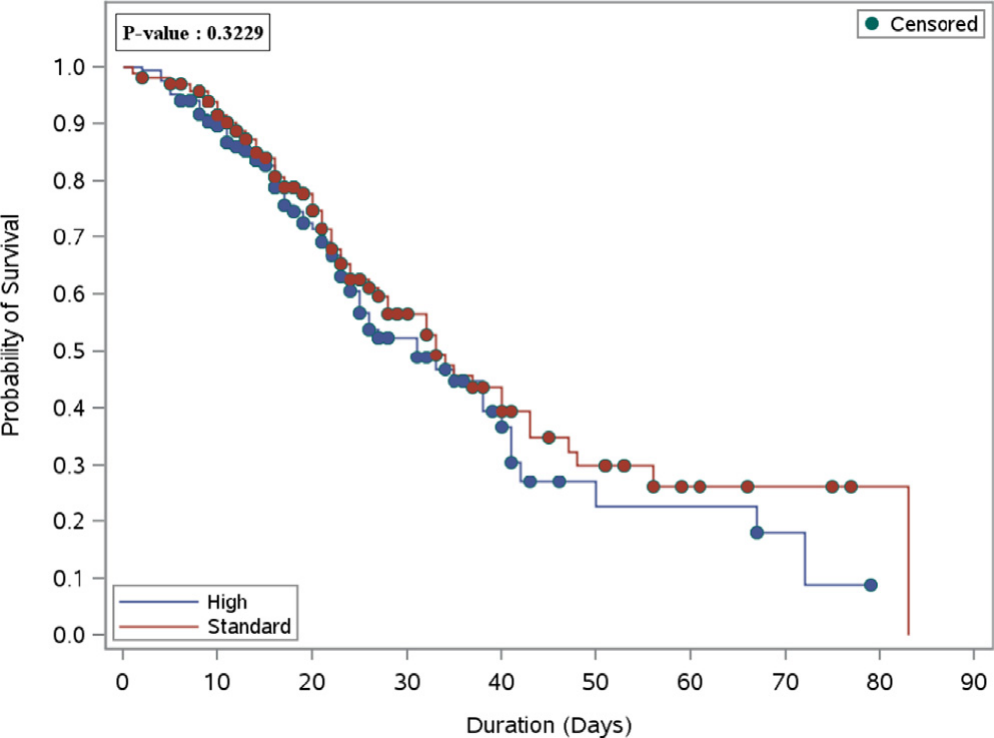
Kaplan Meier Curve for overall survival comparing the escalated "High" dose and standard dosing of VTE prophylaxis during ICU stay - After Propensity score matching.

## 4 Discussion

This two-center retrospective cohort study showed no significant difference in the incidence of VTE and any thrombosis event in patients who received escalated-dose versus standard-dose pharmacological VTE prophylaxis during the ICU stay. However, critically ill patients who received escalated-dose VTE prophylaxis were 3.5 more likely to develop minor bleed compared to patients who received a standard-dose regimen (OR 3.39; 95% CI 1.08–10.61; *P* = 0.04). In December 2020, the national institute of health (NIH) released an interim analysis of three international clinical trials, including more than 1000 critically ill patients with COVID-19 (National Insitue of Health, 2020). The arm of critically ill patients was terminated since those who received therapeutic doses of heparin showed increased mortality and major bleeding compared to controls (National Insitue of Health, 2020). Therefore, newer recommendations and guidelines are against using VTE prophylaxis dose escalation in critically ill patients (Al-Samkari et al., 2021, Cuker et al., 2021, Klok et al., 2020).

There was no statistical difference in the incidence of VTE between the two regimens witnessed in this study (OR 0.75; 95% CI 0.16–3.38; *P* = 0.70). Similarly, the INSPIRATION study did not find a significant difference between ICU patients who received intermediate-dose versus the standard-dose group (OR 0.93; 95% CI 0.37–2.32; *P* = 0.94) (Sadeghipour et al., 2021). Inconsistent with these results, another retrospective study including 852 ICU patients demonstrated no statistical difference in the reported incidence of VTE in patients who received therapeutic anticoagulation dose compared to the prophylaxis dose (*P* = 0.4) (ATTACC Investigators et al., 2021). Even though the median PADUA score for patients included in our study in both groups was five, representing a patient population at high risk for VTE, the VTE incidence in both groups was lower than previously reported incidence in ICU patients (3.4% to 31%) (Al-Samkari et al., 2021, Klok et al., 2020, Sadeghipour et al., 2021).

It is important to highlight that our findings did not observe a superiority of intermediate–dosing to the standard prophylactic dosing regimen in critically ill patients with COVID-19. However, previous studies report that these patients have a higher incidence of VTE development. Accordingly, one can potentially conclude that several factors are of importance in these patients besides the dose of anticoagulation. Such as the timing of anticoagulation initiation and the degree of inflammation and coagulation (disease severity) at the time therapy is initiated and commenced. Moreover, the heterogenicity among the available studies considering those two elements might explain those contradictive findings in terms of the beneficial efficacy of higher prophylactic dosing in these populations.

Our study showed no differences in major bleeding rates similar to the INSPIRATION study (Sadeghipour et al., 2021). It is worth mentioning that the INSPIRATION study noticed severe thrombocytopenia in patients receiving intermediate dosing compared to none in those who received standard dose (Sadeghipour et al., 2021). Several other reports showed no increase in the risk of bleeding in patients receiving high pharmacological thromboprophylaxis doses (Jonmarker et al., 2020, Lavinio et al., 2021, Tacquard et al., 2021). However, in our study, the rate of minor bleeding was significantly higher in the escalated dose group (7.5%) than (2.3%) in the standard group. Our study observed this rate despite that aPTT, d-dimer, fibrinogen, and platelet levels were not significantly different between the two groups. In contrast to our findings, a previous retrospective study of 152 critically ill patients with COVID-19 reported a lower minor bleeding rate in the patients who received high dose thromboprophylaxis (2.7%) compared to the low dose group at 4.5% (Jonmarker et al., 2020). The mechanism of higher prophylactic dosing causing harm is still uncertain. Safety on anticoagulation regimen is beyond the increasing bleeding incidence hypothesis. Previous studies report the autopsy findings of alveolar hemorrhage besides the presence of micro-thrombosis. Thus, we hypothesize that patients with higher inflammatory and hyper-coagulopathy markers (disease severity) are potentially at risk for major bleeding events propagated by the anticoagulation intensity.

Our study found no difference in the in-hospital mortality, 30-day mortality, and the need for MV. On the other hand, two previous retrospective studies reported a significant reduction in the in-hospital mortality, 28-day mortality, and the need for MV (Jonmarker et al., 2020, Roomi et al., 2021). These contradicting findings may be due to the variation in disease severity among the included patients in the two groups. Even though patients who received standard doses had higher metrics for disease severity, such as higher scores (i.e., APACHE II and SOFA scores), all these proposed confounders were further adjusted for baseline differences in our study.

Previous reports of critically ill patients with COVID-19 infection showed an increased incidence of new atrial fibrillation (AF) ranging from 10 % to 22% (Abrams et al., 2020, Wang et al., 2020b). This observation may be attributed to several proposed mechanisms, such as the endothelial dysfunction increases oxidative stress and proinflammatory cytokines, which further produces excessive reactive oxygen species that are probably involved in the atrial oxidative injury (Long et al., 2021, Pober and Sessa, 2007, Teuwen et al., 2020). As an exploratory secondary outcome, we observed a similar rate of new-onset AF between recipients of standard and escalated prophylaxis dosing of 9.9% vs. 12.7%, respectively. Further evaluations for dosing, timing, and mode of thromboprophylaxis in critically ill patients with COVID-19 and new-onset AF are warranted (Long et al., 2021, Pober and Sessa, 2007, Teuwen et al., 2020).

This study's main limitation is the retrospective observational nature leaving residual confounding despite propensity score matching. Furthermore, the decision to prescribe standard or escalated-dose pharmacological VTE prophylaxis to patients with COVID-19 was guided by the institutional and the MOH treatment protocols, which faced a dynamic change as evidence emerged over time (Saudi Ministry of Health, 2021b, Saudi Ministry of Health, 2020). Moreover, confirmation of clinically evident VTE by routine screening was a culprit to limit exposure in some cases. Lastly, VTE prophylaxis dose adjustment based on patient characteristics might have reflected the results.

The optimal pharmacological thromboprophylaxis dosing regimen remains uncertain as studies varied in terms of the patient population, type, dose of anticoagulation, and inclusion or exclusion criteria. Several studies are still ongoing looking at various anticoagulation strategies (Neal Mathew, n.d.). Until additional information, the selection of pharmacological VTE prophylaxis intensity will remain driven by patients related factors. The balance between the patients’ thrombosis and bleeding risk is warranted to guide further pharmacological thromboprophylaxis intensity. Perhaps, critically ill patients with COVID-19 may benefit from targeting multiple pathways involved in the pathogenesis of the immune system, such as cytokines-targeted therapy or the utilization of non-heparin products (direct thrombin inhibitors), which deserve further studies.

## 5 Conclusion

Our two-center retrospective cohort study concurs with previous studies' findings that escalated-dose pharmacological VTE prophylaxis in critically ill patients with COVID-19 was not associated with VTE or mortality benefits but was linked to an increased risk of minor bleeding. This study supports previous evidence regarding the optimal dosing for VTE pharmacological prophylaxis regimen incritically ill patients with COVID-19.

## 6 Disclaimer

The contents of this manuscript are solely the authors’ views and may not be understood or quoted as being made on behalf of or reflecting the position of the Saudi Food and Drug Authority.

## Declaration of Competing Interest

The authors declare that they have no known competing financial interests or personal relationships that could have appeared to influence the work reported in this paper.
