# Supplementary material for: Comparison between standard Vs. Escalated dose venous thromboembolism (VTE) prophylaxis in critically ill patients with COVID-19: A two centers, observational study
Source: Saudi Pharm J. 2022 Feb 3;30(4):398–406. doi: 10.1016/j.jsps.2022.01.022 (PMC8812085; doi:10.1016/j.jsps.2022.01.022)
Supplement: Supplementary data 1 [file mmc1.docx]

**Table S1 Comorbidities of the patients admitted to the intensive care unit (ICU) with COVID-19 using anticoagulation for thromboprophylaxis before and after matching**

|  | **Before Propensity score Matching** | | | | | **After Propensity score Matching** | | | |
| --- | --- | --- | --- | --- | --- | --- | --- | --- | --- |
|  | **Overall (565)** | **Standard dose (N=380)** | **Escalated dose (N=185)** | ***P* value** | **Overall (352)** | | **Standard dose (N=176)** | **Escalated dose  (N=176)** | ***P* value** |
| **Dyslipidemia (DLP)** | 126 (22.8) | 90 (24.1) | 36 (20) | 0.2781^^ | 80 (23.1) | | 46 (26.4) | 34 (19.7) | 0.1336^^ |
| **Diabetes mellitus (DM)** | 332 (60) | 229 (61.4) | 103 (57.2) | 0.3480^^ | 199 (57.3) | | 99 (56.9) | 100 (57.8) | 0.8644^^ |
| **Hypertension (HTN)** | 309 (55.9) | 215 (57.6) | 94 (52.2) | 0.2292^^ | 188 (54.2) | | 97 (55.7) | 91 (52.6) | 0.5565^^ |
| **Acute Coronary Syndrome (ACS), n (%)** | 6 (1.1) | 6 (1.6) | 0 (0) | 0.0867** | 4 (1.2) | | 4 (2.3) | 0 (0.0) | 0.0443** |
| **Asthma, n (%)** | 52 (9.4) | 32 (8.6) | 20 (11.2) | 0.3337^^ | 38 (11.0) | | 19 (10.9) | 19 (11) | 0.9850^^ |
| **Atrial fibrillation (AFib or AF), n (%)** | 6 (1.1) | 6 (1.6) | 0 (0) | 0.0871** | 1 (0.3) | | 1 (0.6) | 0 (0) | 0.3180** |
| **Chronic obstructive pulmonary disease (COPD)** | 7 (1.3) | 3 (0.8) | 4 (2.2) | 0.1622** | 5 (1.4) | | 1 (0.6) | 4 (2.3) | 0.1745** |
| **Cancer (any type)** | 19 (3.5) | 11 (3) | 8 (4.5) | 0.3716^^ | 12 (3.5) | | 5 (2.9) | 7 (4.1) | 0.5567^^ |
| **Chronic kidney disease (CKD)** | 52 (9.5) | 45 (12.1) | 7 (3.9) | 0.0020^^ | 17 (4.9) | | 10 (5.7) | 7 (4) | 0.4630^^ |
| **Coronary artery bypass grafting (CABG)** | 13 (2.4) | 9 (2.4) | 4 (2.2) | 0.8900** | 9 (2.6) | | 5 (2.9) | 4 (2.3) | 0.7422** |
| **Heart failure (HF)** | 35 (6.4) | 25 (6.7) | 10 (5.6) | 0.5934^^ | 18 (5.2) | | 8 (4.6) | 10 (5.8) | 0.6194^^ |
| **Hypothyroidism** | 30 (5.4) | 21 (5.7) | 9 (5) | 0.7487^^ | 15 (4.3) | | 7 (4.0) | 8 (4.6) | 0.7830^^ |
| **Ischemic heart disease (IHD)** | 37 (6.8) | 26 (7.1) | 11 (6.1) | 0.6877^^ | 21 (6.1) | | 10 (5.8) | 11 (6.4) | 0.8325^^ |
| **Liver disease (any type)** | 10 (1.8) | 6 (1.6) | 4 (2.2) | 0.6054** | 5 (1.4) | | 1 (0.6) | 4 (2.3) | 0.1724** |
| **CVA (Stroke)** | 31 (5.6) | 28 (7.6) | 3 (1.7) | 0.0049^^ | 17 (4.9) | | 14 (8.1) | 3 (1.7) | 0.0062^^ |
| **Venous thromboembolism (VTE) (PE_DVT)** | 3 (0.5) | 1 (0.3) | 2 (1.1) | 0.2080** | 2 (0.6) | | 0 (0) | 2 (1.2) | 0.1549** |
| *T Test / ^ Wilcoxon rank sum test is used to calculate the P-value.  ^^ Chi square/ ** Fisher’s Exact teat is used to calculate P-value. | | | | | | | | | |
